# Bidirectional Neoadjuvant Chemotherapy for Patients with Gastric Cancer and Synchronous Peritoneal Metastases (GCPMs): Results of a Western Phase II Study

**DOI:** 10.3390/jcm14186518

**Published:** 2025-09-16

**Authors:** Daniele Biacchi, Marco Angrisani, Vincenzo Picone, Daniele Scuto, Maria Gloria Gallotti, Fabio Accarpio, Franco Iafrate, Giorgio Masci, Immacolata Iannone, Alessandra Spagnoli, Paolo Sammartino

**Affiliations:** 1 Department of Surgery Cytoreductive Surgery and HIPEC Unit, Pietro Valdoni, Umberto I Policlinico di Roma, 00161 Rome, Italyimmacolata.iannone@uniroma1.it (I.I.);; 2 Department of General Surgery and Transplantation Unit, San Camillo Forlanini Hospital, 00152 Rome, Italy; 3 Department of Radiological, Oncological and Pathological Science, Sapienza University of Rome, 00161 Rome, Italy; 4 Department of Public Health and Infectious Diseases, Sapienza University of Rome, 00161 Rome, Italy

**Keywords:** gastric cancer, peritoneal surface malignancies, cytoreductive surgery, peritoneal metastases, hyperthermic intraperitoneal chemotherapy, pressurized intraperitoneal aerosol chemotherapy

## Abstract

**Background:** The outcomes of patients with peritoneal metastases from gastric cancer (GCPMs) remain dismal, with an overall survival (OS) of less than 1 year. Approaches reported from East Asia include normothermic intraperitoneal systemic chemotherapy, aimed at downstaging the disease, allowing an R0 resection. This is the first Western study evaluating a bidirectional regimen in a neoadjuvant setting of GCPMs. This phase II study evaluates the tolerability, efficacy and conversion surgery rate. **Methods:** Patients with PCI < 13 without ascites or HER2 overexpression and no extraperitoneal spread were enrolled starting in January 2018. After staging laparoscopy combined with PIPAC (cisplatin + doxorubicin), NIPS began following Yonemura’s schedule: cisplatin (30 mg/m^2^) + docetaxel (30 mg/m^2^), intraperitoneally (day 1); capecitabine 1000 mg/m^2^, orally (days 2–15); and cisplatin (30 mg/m^2^) + docetaxel (30 mg/m^2^), intravenous (day 8). After three cycles, patients with no progressive disease and negative peritoneal cytology underwent cytoreductive surgery (CRS) combined with hyperthermic intraperitoneal chemotherapy (HIPEC). Three additional NIPS cycles were reserved for patients who underwent surgery. **Results:** Among the 25 treated patients with 17.3-month (95%CI: 10.4; NA) OS, no adverse events (CTCAE) ≥ G3 arose. With a 52% conversion surgery rate, 13 patients underwent CRS combined with HIPEC (cisplatin 100 mg/m^2^), 10 with CC0 status 3 with CC experienced no operative mortality, and major complications rated Clavien–Dindo IIIB occurred in 2 patients (15.4%). The median OS for patients undergoing surgery was 26 (95%CI: 23.1; NA) months, with progression-free survival of 20 (95%CI: 16.7–NA) months. **Conclusions:** NIPS is safe and effective. The conversion rate in our Western patients is comparable to that reported in Eastern Asian countries.

## 1. Introduction

Gastric cancer (GC) remains a significant clinical challenge, ranking fifth in terms of both incidence and mortality worldwide [1]. In recent years, along with a decrease in the incidence of GC linked to environmental factors (intestinal type), in Europe, in the USA, and even in Eastern countries, a relative increase in more aggressive histotypes, such as poorly cohesive neoplasms, has been observed, leading to a higher prevalence of GC with peritoneal metastases (GCPMs) at diagnosis and an increasingly younger age of onset [2,3,4,5,6]. In a recent report, up to 32% of patients with GC who underwent staging laparoscopy before treatment had peritoneal involvement [7]. Patients affected by GCPMs pose a complex therapeutic challenge. These patients usually present at diagnosis with a heavy peritoneal burden, and they tend to be micro-satellite stable, HER2 negative and with low expression of PD-L1, so they are less likely to benefit from immune or targeted therapies [8]. The current standard of care for patients with synchronous GCPMs in Western countries is systemic chemotherapy (SC); however, the outcomes, depending on the response to therapy or histological subtype, remain poor, with median overall survival (OS) of 7.5 months and median time to treatment failure (TTL) of 4.6 months [9,10,11]. Recently, a series of innovative treatments have emerged, based on the integration of SC with forms of locoregional chemotherapy [12]. Given the low penetration of SC into the peritoneal cavity [13], the delivery of intraperitoneal chemotherapy reduces the clearance of the drugs (plasma–peritoneal barrier), reaching higher concentrations and allowing prolonged exposure of metastatic disease with limited systemic toxicity [14]. The first adoption of intraperitoneal chemotherapy combined with hyperthermia (HIPEC) was associated with surgery, dating back to the late 1980s and reported by Japanese authors, both as a treatment and as a prevention of peritoneal spread in GC [15,16]. Given the low rate of GCPM patients who can be surgically treated with radical intent at the onset, over time, intraperitoneal chemotherapy combined with SC (bidirectional approach) has been employed primarily by authors from the Far East as a neoadjuvant therapy [17]. Three methods of intraperitoneal chemotherapy delivery with this aim are currently in use. Catheter-based normothermic intraperitoneal chemotherapy administered to ambulatory patients (NIPS procedure, according to Yonemura) is the oldest and most widely reported technique [18]. Emerging techniques, such as laparoscopic HIPEC (LS-HIPEC) and pressurized intraperitoneal aerosol chemotherapy (PIPAC), are currently being evaluated to expand both curative and palliative therapeutic options [19,20]. Currently, numerous phase II studies and four randomized trials appear to demonstrate the efficacy of locoregional chemotherapy combined with systemic treatment, compared to systemic chemotherapy alone, in terms of the disease reduction, conversion rates to curative-intent surgery, and, ultimately, overall survival [17,21,22,23,24,25]. The increase in available therapeutic options, along with improvements in patient selection criteria, has opened up new perspectives in this clinical scenario. Patients with a limited tumor burden (oligometastatic GC) who undergo an aggressive, multimodal, and integrated approach—combining systemic chemotherapy with locoregional procedures—may, in the event of a therapeutic response, be considered for radical-intent surgery (conversion surgery) [26,27] with cytoreductive surgery (CRS) combined with HIPEC. The aim of this study is to evaluate the efficacy of a bidirectional neoadjuvant approach based on Yonemura’s protocol in patients with GCPMs undergoing staging laparoscopy combined with PIPAC. The endpoints include assessing the treatment safety, conversion surgery rate, overall survival (OS), and progression-free survival (PFS).

## 2. Materials and Methods

### 2.1. Patient Eligibility and Study Design

This study was designed as a single-center, phase II study. Patients considered for the study were those with GC according to the last AJCC classification [28] (including Siewert III tumors) with synchronous macroscopic PMs (excluding patients with only positive cytology), without other metastatic sites, no her2/neu overexpression or unsuitable for immunotherapy.

The eligibility criteria included (1) histologically proven GC with synchronous PMs diagnosed by laparoscopy in patients not undergoing previous therapeutic interventions; (2) aged between 18 and 70 years; (3) performance status (Eastern Cooperative Oncology Group) of 0–1; (4) Peritoneal Cancer Index (PCI) < 13 at staging laparoscopy (29); (5) adequate cardiac, renal (serum creatinine level within the upper limit of the normal), hepatic (total serum bilirubin level < 2.0 mg/dL and serum transaminases < 100 UI) and bone marrow function (leukocyte count 3000–12.000/mm^3^, hemoglobin > 8.0 g/dL and platelet count > 100.000/mm^3^); (6) able to have an adequate caloric intake and expected survival period of >3 months; and (7) able to provide informed written consent. The exclusion criteria were (1) ascites; (2) presence of distant metastases (liver, lung, brain, bone) or paraaortic and/or extra-regional lymphatic spread; (3) other cancer diagnoses in the last 5 years (excluding cutaneous basalioma or preinvasive cervical carcinoma); and (4) pregnancy or breastfeeding status or other systemic illness preventing inclusion in the protocol. This study was carried out in accordance with the Declaration of Helsinki, was approved by the Department of Surgery Institutional Review Board (A03/23) and was supported by a grant from Sapienza University of Rome (RG12117A807F5D85).

**Figure 1:**
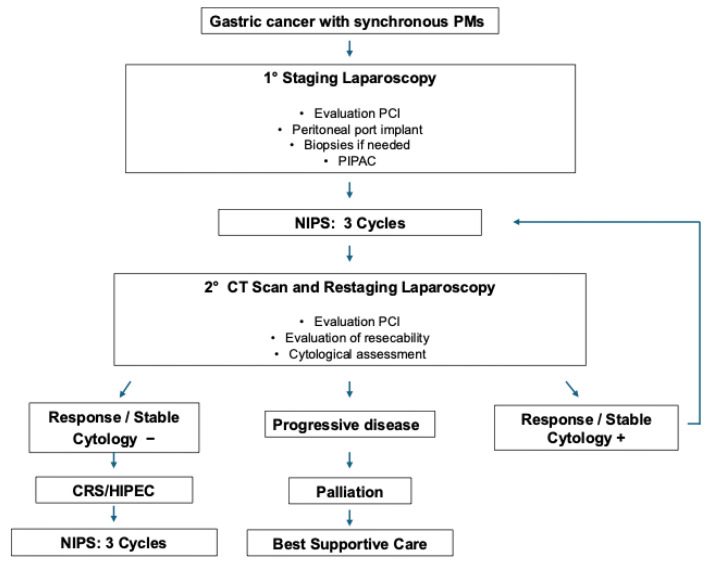
Algorithm followed in the study. PMs, peritoneal metastases; PCI, Peritoneal Cancer Index; CT scan, computed tomography; PIPAC, pressurized intraperitoneal aerosol chemotherapy, normothermic intraperitoneal and systemic chemotherapy; CRS, cytoreductive surgery; HIPEC, hyperthermic intraperitoneal chemotherapy.

As shown in Figure 1, according to the algorithm followed in the study, patients with GCPMs after diagnosis and a total body CT scan excluding other sites of metastatic spread underwent a first staging laparoscopy to evaluate the extent of peritoneal involvement (PCI) and quantify the amount of ascites considered in the inclusion criteria. Patients meeting the inclusion criteria were enrolled in the study, and after surgical implanting of a peritoneal port device (Celsite Peritoneal 15 F) under laparoscopic assistance [29] with the port tip placed in the cul-de-sac, underwent the PIPAC procedure with cisplatin and doxorubicin (cisplatin 10.5 mg/mq doxorubicin 2.1 mg/mq). Once the surgical wounds had healed, a bidirectional chemotherapy (NIPS) started, and after 3 cycles, patients underwent a CT scan and restaging laparoscopy. Peritoneal lesions were sampled, and aspiration of peritoneal fluid was performed for cytological assessment. Patients considered responders or with stable disease at the restaging laparoscopy, no malignant cells on cytology with no progression of disease on CT imaging and resectable disease, and who were fit enough to undergo surgery become candidates for conversion surgery. Patients considered responders or with stable disease but with positive peritoneal cytology were submitted to further NIPS cycles until the disappearance of this indicator would make them eligible for surgery. Patients with progressive disease left the study toward palliative procedures or best supportive care. Patients who underwent CRS combined with HIPEC received after surgery 3 further NIPS cycles as adjuvant treatment.

### 2.2. Treatment Schedule: Bidirectional Chemotherapy (NIPS)

**Figure 2:**
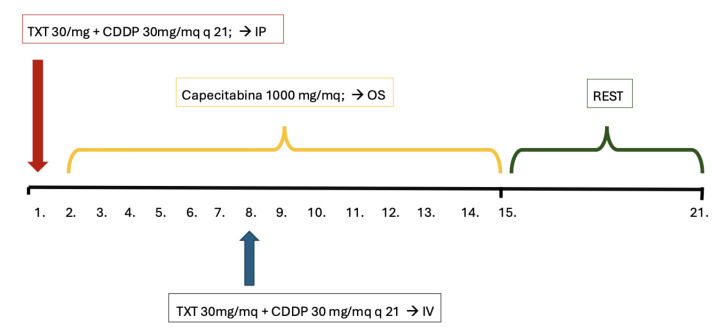
Schematic illustration of one course of bidirectional systemic and intraperitoneal chemotherapy for peritoneal metastasis from gastric cancer. IV, intravenous; OS, oral; IP, intraperitoneal.

All patients received bidirectional chemotherapy in the Medical Oncological Department and a series of 3-week cycles of NIPS was performed. Capecitabine was administered orally (1000 mg/m^2^), on days 2 to 14 for 14 consecutive days, followed by 7 days of rest. Taxotere and cisplatin were administered through the peritoneal port system (i.p.), diluted in 500 mL of saline solution at a dose of 30 mg/m^2^, each on day one. The same doses of taxotere and cisplatin were administered intravenously (i.v.) on day 8 after standard premedication. Therapy was stopped prematurely for unacceptable toxicity, disease progression or at the patient’s request (Figure 2).

### 2.3. Surgical Procedure

After four weeks from the last NIPS cycle, patients considered resectable according to the CT scan and restaging laparoscopy, and who fulfilled the inclusion and exclusion criteria for the study, underwent surgery. The cytoreduction procedures included, after intraoperative PCI evaluation at the beginning of surgery, in every patient total gastrectomy with D2 lymphadenectomy along with excision any way of target structures of peritoneal spread, such as the greater and lesser omentum, cecal appendix, round and falciform ligament of the liver, and, in postmenopausal patients, bilateral adnexectomy. Each cytoreduction procedure included peritonectomy procedures and visceral resections according to the extent of the disease, with the goal of obtaining a completeness of cytoreduction (CC) score of CC 0/CC1 [30]. Surgical procedures considered radical were followed by an HIPEC with closed technique with cisplatin 100 mg/m^2^, in a maximum of 5 L of saline. The perfusate was delivered into the abdomen at 40–42° over 60 min at a flow rate of 800–1000 mL/min.

### 2.4. Evaluation of Outcomes

The primary endpoints of this study were the conversion surgery rate and treatment-related adverse effects in all patients enrolled in the study. After each cycle of treatment, physical examination, laboratory analysis (adequate cardiac, renal, hepatic and bone marrow function) and chest radiography were performed. Drug-induced toxicity was graded according to the National Cancer Institute Common Terminology Criteria for Averse Events (CTCAE version 4.0) [31]. Secondary endpoints included OS and progression-free survival (PFS); survival was calculated from the time of enrolment in the study to the time of death due to any cause or diagnosis of disease progression. Tumor responses were evaluated after every three cycles by CT scan and categorized according to the Response Evaluation Criteria in Solid Tumors (RECIST) revised guideline version [32]. During restaging laparoscopy and at laparotomy for the patients who were candidates for CRS combined with HIPEC, the change in the PCI score was evaluated. In patients who underwent CRS combined with HIPEC, surgery-related morbidity was evaluated according to the Clavien–Dindo Classification [33]; minor morbidity was defined as grade I–IIIA, whereas major morbidity was defined as grade IIIB–V. Tumor responses in resected specimens were assessed according to the Peritoneal Grade Regression Score (PRGS) [34]. All patients who underwent surgery received three additional cycles of adjuvant bidirectional chemotherapy, following the same regimen used during the neoadjuvant phase.

### 2.5. Statistical Analysis

Patient characteristics were described using the median and interquartile range (IQR) for continuous variables, and as absolute frequencies and percentages for categorical variables. Overall survival (OS) was defined as the time from the date of enrollment to death from any cause. Patients without an OS event were censored at the date they were last known to be alive. Progression-free survival (PFS) was defined as the time from the date of enrollment to the first documented disease recurrence or death from any cause. Patients without a PFS event were censored at the date of their last disease assessment. The PFS and OS probabilities were estidespmated using the non-parametric Kaplan–Meier method and presented graphically. A *p*-value of less than 0.05 was considered statistically significant. Confidence intervals were calculated at the 95% level. All the statistical analyses were performed using R software (version 4.5.0, R Foundation for Statistical Computing, Vienna, Austria).

## 3. Results

**Figure 3:**
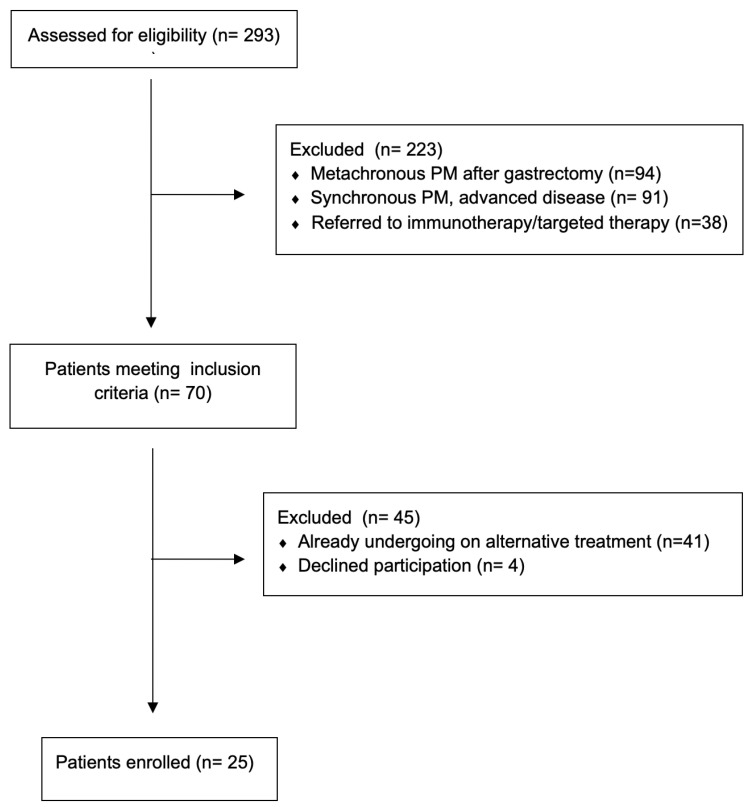
CONSORT flow diagram of patient screening and enrollment.

During the study period (January 2018–December 2024), 293 patients affected by peritoneal metastases from gastric cancer were evaluated in our center. A total of 94 patients were affected by metachronous peritoneal metastases after a previous gastrectomy for cancer, 91 had synchronous GCPMs but with disease extension beyond the eligibility criteria for this study, while 41 patients with synchronous GCPMs, and although they met the criteria for inclusion in the study, they had already started systemic chemotherapy at the time of evaluation. Considering also 38 patients with synchronous GCPMs suitable for immune or target therapy, and excluding 4 patients who refused to participate, 25 patients were finally enrolled in the study (Figure 3).

Patient characteristics are presented in Table 1. Following the initial PIPAC procedure, a total of 86 cycles of bidirectional normothermic chemotherapy (NIPS) were administered across the cohort, with a median of 3 cycles per patient. Two patients developed parietal fluid collections in the immediate postoperative period following PIPAC, which led to a delay in the initiation of NIPS. As shown in Figure 1, peritoneal cytology was not performed at the initial diagnostic laparoscopy, but at restaging laparoscopy after neoadjuvant therapy, it was systematically assessed to confirm eligibility for CRS.

Treatment was generally well tolerated, and all the NIPS-related adverse events were classified as grade < 3 according to the Common Terminology Criteria for Adverse Events (CTCAE). No intraperitoneal infections or chemoport-related complications were observed. At the end of the neoadjuvant treatment and after restaging, 13 patients (52%) underwent surgery: 12 after three cycles of NIPS and 1 after six cycles. The remaining 12 patients (48%) showing disease progression during treatment occurring after a median of four NIPS cycles, left the study toward palliative procedures or best supportive care. Disease progression involved both the peritoneum and liver in five cases, the liver alone in three cases, the peritoneum alone in two cases, the mediastinum in one case, and both the mediastinum and liver in one additional patient.

**Figure 4:**
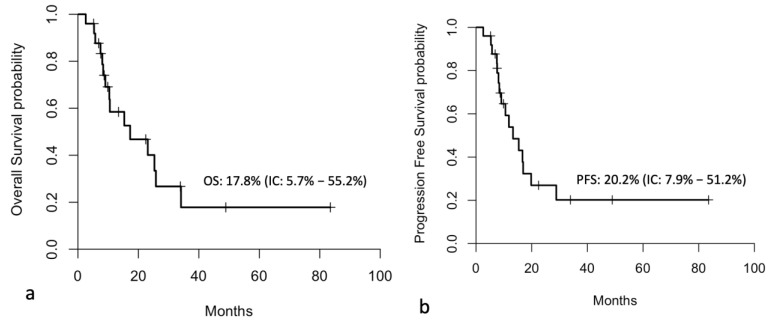
Kaplan–Meier curves showing overall survival (**a**) and progression-free survival (**b**) in all enrolled patients.

The median follow-up was 33.9 months (range: 5.21–83.51 months). The median overall survival (OS) for the entire cohort was 17.3 months, while the median progression-free survival (PFS) was 13.3 months (Figure 4).

**Figure 5:**
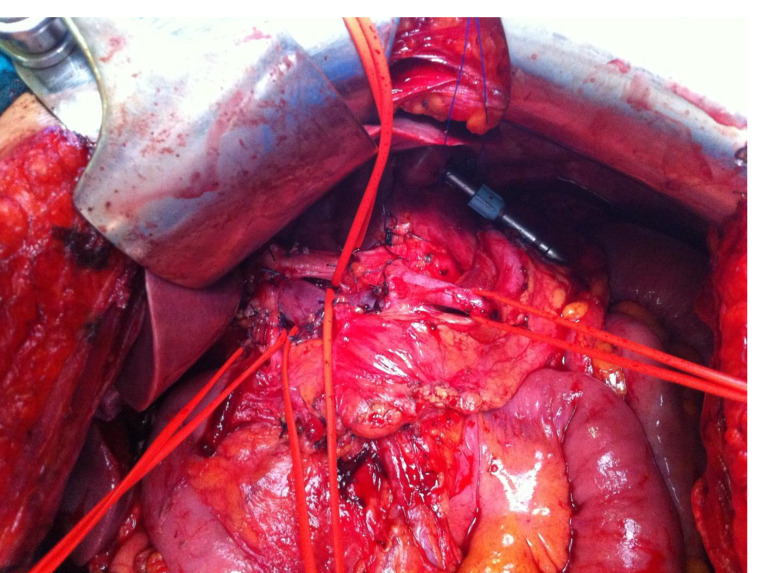
Surgical field showing extended D2 lymphadenectomy performed according to the Japanese Gastric Cancer Treatment Guidelines (6th edition).

Conversion surgery was performed in 13 patients (52%) who underwent cytoreductive surgery (CRS) combined with hyperthermic intraperitoneal chemotherapy (HIPEC) using cisplatin (100 mg/m^2^). Ten patients had a tumor involving the proximal third of the stomach and all showed evident involvement of the gastric serosa. In all 13 patients who underwent surgery, a total gastrectomy and D2 lymphadenectomy according the Japanese Gastric Cancer Treatment Guidelines (6th edition) were performed [35], with dissection of stations 1–7 plus 8a, 9, 11p, 11d, 12a and 10 when splenectomywa needed (Figure 5).

Cytoreductive surgery was performed according to the peritonectomy procedures proposed by PH Sugarbaker [36], with resection regardless of their involvement in all the target structures of peritoneal dissemination, including bilateral adnexectomy in female patients. No postoperative mortality was observed. Major complications occurred in two patients, both due to hemoperitoneum requiring reoperation. Complete cytoreduction (CC-0) was achieved in 10 patients (Table 2).

**Figure 6:**
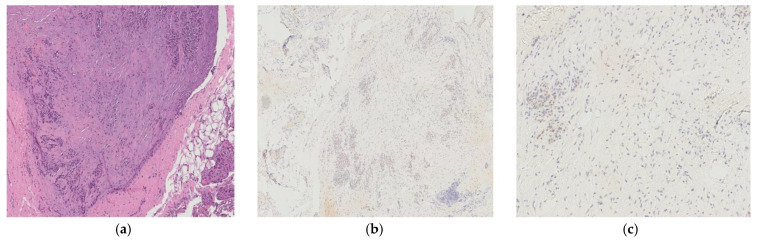
Histological response in a resected peritoneal lesion from a patient undergoing CRS and HIPEC. H&E staining (**a**) shows predominant fibrosis with scattered residual tumor glands. CDX2 immunostaining at low (**b**) and high (**c**) magnification highlights focal residual neoplastic cells within fibrotic stroma, consistent with PRGS grade 2.

In all the resected cases, the pathological response to neoadjuvant chemotherapy was classified as PRGS grade 2, characterized by fibrosis and necrosis predominating over residual tumor tissue (Figure 6).

**Figure 7:**
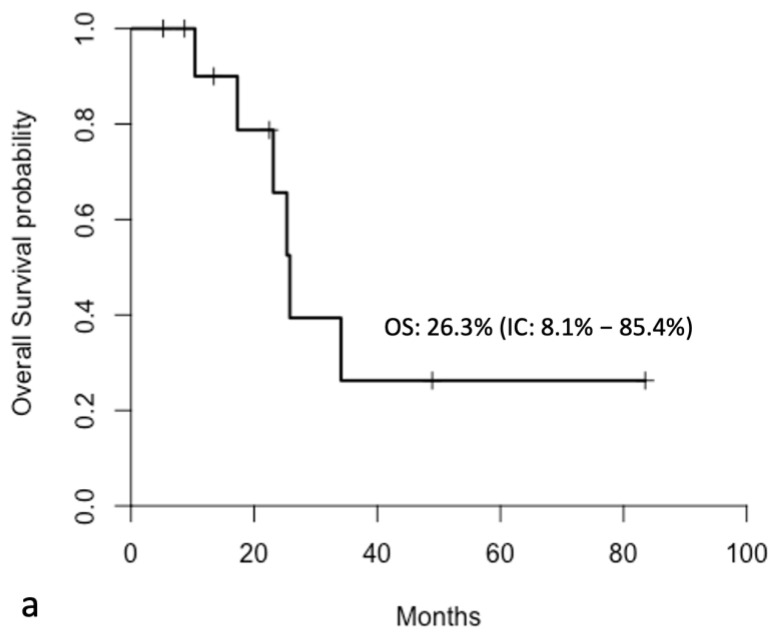

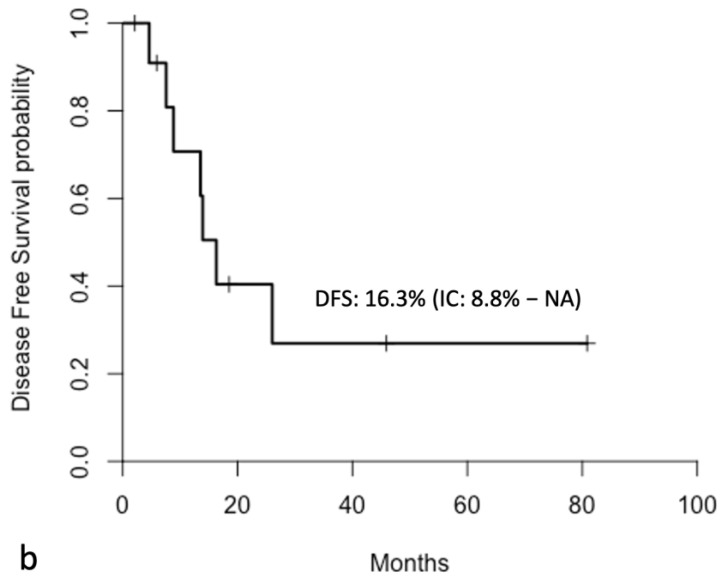
Kaplan–Meier curves showing **overall survival** (**a**) and **progression-free survival** (**b**) in patients undergoing NIPS + CRS.

A reduction in PCI was observed only in patients who underwent surgery (n = 13), with a decrease from a median of 12 [IQR(7–12)] at baseline to 6 [IQR (4.5–10)] after NIPS. No reliable post-treatment PCI assessment was possible in the 12 patients who progressed and did not undergo surgical exploration. All the patients who underwent surgery also received three additional cycles of adjuvant NIPS with the same regimen used in the neoadjuvant phase. At the time of the last follow-up, 6 of the 13 resected patients were alive and disease-free, 1 patient was alive with peritoneal recurrence, and 6 patients had died. Among the deceased patients, three died due to isolated peritoneal recurrence, one due to combined peritoneal and hepatic recurrence, one due to central nervous system recurrence, and one due to pleural and mediastinal relapse. Among the patients who underwent surgery, the median overall survival (OS) was 26 months and the median progression-free survival (PFS) was 20 months (Figure 7).

## 4. Discussion

The results we obtained are similar to those recently reported in the literature, both in terms of the oncologic outcomes and in the percentage of patients converted to radical surgery following bidirectional treatment for synchronous GCPMs [22,37,38,39,40,41,42,43,44,45,46]. The results from the REGATTA trial conducted in Japan and Korea showed that in patients with stage IV gastric cancer (70% of whom had peritoneal involvement), palliative surgery does not offer a survival benefit over chemotherapy alone [47], thus emphasizing the key role of neoadjuvant therapy in the treatment paradigm for these patients. Despite the frequent presentation of peritoneal dissemination at diagnosis in gastric cancers [48], data from large series have demonstrated that, when appropriate selection criteria are applied and oligometastatic disease is identified, in patients considered responders to neoadjuvant treatments, radical surgery may be achievable in many cases, yielding significant prognostic advantages [26,27,49]. In choosing a neoadjuvant treatment for synchronous GCPMs, pathological review, biomarker testing, and molecular characterization of tumor tissue play a crucial role. In our cohort, we excluded patients eligible for targeted or immunotherapy. Every diagnostic specimen was tested for HER2, MSI/MMR status, and PD-L1 expression. Moreover, HER2 overexpression, microsatellite instability, and CPS > 5 are more frequently observed in intestinal-type tumors [50,51,52], which are less prone to peritoneal spread. This effectively deprives diffuse or poorly cohesive gastric cancers—which are more likely to metastasize to the peritoneum—of key therapeutic options. The management of gastric cancer with synchronous peritoneal metastases remains one of the most complex challenges in gastrointestinal oncology. Systemic chemotherapy (oral or intravenous) generally provides disappointing results [3]. Intraperitoneal delivery has been explored to maximize the local drug concentration while minimizing systemic toxicity. There are, however, major geographical differences in the adoption of these strategies. In Western countries, intraperitoneal therapy is typically limited to intraoperative HIPEC or palliative PIPAC. Conversely, a recent meta-analysis by Boshier et al. [17] reported 28 studies from East Asia involving 1649 patients—80% with synchronous GCPMs—treated with catheter-based (normothermic) intraperitoneal chemotherapy as part of a bidirectional regimen alongside concurrent systemic chemotherapy Within this context, our study represents one of the few Western prospective experiences [53,54] adopting a structured bidirectional approach, preceded by PIPAC and followed by cytoreductive surgery and HIPEC. In our study, treatment was well tolerated: no ≥G3 adverse events occurred during the NIPS phase, and no intraperitoneal-access-related complications were observed. This is consistent with the good tolerability reported by Saito et al. [46], who evaluated the combination of intraperitoneal paclitaxel and SOX, showing an excellent safety profile even in prolonged treatments of up to 16 cycles. Similarly, Cho et al. [43], using intraperitoneal docetaxel combined with capecitabine and cisplatin, reported manageable toxicities, although with a higher cumulative incidence of abdominal pain in later treatment cycles. The most relevant outcome in our series was the conversion surgery rate, which reached 52%, with R0/CC-0 resection achieved in 77% of the resected patients. These results are consistent with those reported in major Asian studies. Saito et al. [46] reported a surgery rate of 45% with a median OS of 25.8 months among resected patients. Chia et al. [55] observed a surgery rate of 36% and a median OS of 24.2 months in operated patients. Kang et al. [56], although reporting a lower conversion rate (23%), also highlighted the benefit of IP chemotherapy over historical controls treated with systemic chemotherapy alone. Some aspects of the surgical strategy for these patients should be emphasized. The first point concerns the role of cytology assessed at the time of restaging. The prognostic role of a positive cytology finding in the presence or absence of macroscopically evident peritoneal pathology is quite debated, as according to some, this aspect could be controlled oncologically with extensive intraoperative lavage, with the adoption of HIPEC at the time of surgery or with adjuvant systemic chemotherapy [57,58,59]. We considered cytological conversion a prerequisite for proceeding to surgery. Our approach seems justified based on both the results reported by the Italian Peritoneal Surface Malignancies Oncoteam, which analyzed a cohort of patients undergoing CRS plus HIPEC for synchronous GCPMs [60], and the findings of one of the largest published experiences of bidirectional therapy in this setting. Yonemura et al. [45], through multivariate analysis, demonstrated that the conversion of peritoneal cytology from positive to negative following NIPS is an independent prognostic factor. More recently, the updated guidelines of the Chicago Consensus Working Group on GCPMs [61] have emphasized the prognostic importance of achieving cytological negativity through neoadjuvant treatment, recommending it as a key step in improving patient outcomes. Another important aspect worth emphasizing concerns the surgical technique. Given that we are dealing with cytoreductive procedures for peritoneal surface malignancies, the most critical factor to consider is the need to achieve complete cytoreduction. In our experience, this was successfully obtained in over 70% of cases. Nevertheless, in our opinion, it is essential to underscore the importance of adequately addressing the primary gastric tumor, even in the context of synchronous GCPMs. This issue is often overlooked, particularly with regard to the necessity of performing a total gastrectomy. Although the aforementioned American guidelines recommend mandatory D2 lymphadenectomy, they do not consider the extent of the gastric resection as a decisive factor in patient outcomes, effectively leaving the choice to the surgeon based on the tumor location [61,62]. Conversely, our choice to perform total gastrectomy systematically was based on several considerations. First, the majority of synchronous GCPMs included our series were diagnosed with poorly cohesive gastric cancer, for which wider surgical margins are recommended [63]. Second, in most cases, the tumor involved the upper two-thirds of the stomach, making total gastrectomy anatomically and oncologically appropriate. Finally, total gastrectomy facilitates a more complete and technically straightforward dissection of all the lymph node stations included in a standard D2 lymphadenectomy. Moreover, several recent surgical series in the literature on patients who underwent cytoreduction for CGPMs support this approach, reporting the use of total gastrectomy in 70% to 90% of cases, including the studies by Marano ci devi rimettere il n di biblio, Bonnot PE et al. [64] and Manzanedo I et al. [65]. Another key point in this field concerns the ongoing debate regarding the optimal cutoff value—low versus high—for the Peritoneal Cancer Index (PCI) as the main selection criterion for surgery. Western authors tend to adopt more stringent selection criteria regarding this parameter, generally excluding patients with PCI > 12 from surgery due to the increased difficulty of achieving complete cytoreduction [66,67]. Mariani et al. [68], based on the results of Bonnot et al. [64], proposed a more restrictive PCI threshold in the context of poorly cohesive gastric cancer, recommending surgical access only for patients with PCI < 6. However, it is important to note that in Bonnot et al.’s large and well-characterized cohort, only 50% of patients received neoadjuvant therapy, meaning that in half of the cases, the extent of the peritoneal tumor burden was not subject to potential preoperative downstaging. Conversely, several Asian series employing bidirectional treatment protocols have reported significant downstaging following neoadjuvant therapy. Saito et al. [46] achieved conversion surgery in 50% of patients with baseline PCI between 10 and 20; Kang et al. [56] reported a median PCI reduction of seven points after neoadjuvant treatment; and Yonemura et al. [45] were able to reduce the PCI to <11 in 70% of patients undergoing NIPS. Similarly, in our cohort—despite the predominance of poorly cohesive gastric cancer in 84% of cases—bidirectional treatment, often combined with PIPAC, led to a significant PCI reduction in 52% of patients, enabling inclusion in the study of patients with a PCI as high as 12. Despite these initially high PCI values, resected patients exhibited a homogeneous pathological response (PRGS 2) with macroscopic signs of regression and fibrosis. This supports the observation by Saito and colleagues [46] that the PCI alone does not reliably predict outcomes and that parameters such as cytological conversion and disease stability may represent more appropriate criteria for surgical selection. From this perspective, the biological response to treatment should progressively replace the initial disease burden as the primary decision-making metric. Our protocol also included the early use of PIPAC, similar to what was proposed by Casella et al. [54], in patients eligible for curative intent treatment. In the literature, the use of PIPAC in patients with GCPMs is generally limited to those considered unresectable with a high PCI, and it is most often combined with systemic chemotherapy for palliative rather than curative purposes [69,70].

In our view, standardizing the use of this technique as part of a curative strategy is advisable, particularly as an adjunct to conventional systemic therapy. A relevant example is the failure of the GASTRIPEC-1 trial [71], a randomized study comparing cytoreductive surgery plus HIPEC versus cytoreduction alone following systemic chemotherapy. The trial was prematurely terminated because 55 out of 105 patients experienced disease progression during neoadjuvant systemic therapy, highlighting the limitations of systemic treatment alone in this setting.

Our study has some limitations, including the small sample size and lack of a control arm treated with systemic chemotherapy alone, which precludes direct comparison. No formal power calculation was performed. This reflects our role as a surgical referral center for peritoneal metastases, where many patients (41 in our series) were recruited after having already initiated systemic therapy. Furthermore, patients eligible for targeted or immunotherapies (e.g., HER2-positive, MSI-H/dMMR, or high PD-L1 CPS) were intentionally excluded by the protocol, as the expected outcomes of these modern treatments appear superior to those achievable with an approach that, in Western practice, remains investigational. Notably, 38 such patients were screened but not enrolled.

Lastly, the long accrual period (2018–2024) may have introduced temporal heterogeneity into the diagnostic work-up, perioperative management, and access to locoregional strategies, potentially confounding both the conversion rates and survival outcomes. Nonetheless, in a context of scarce prospective Western data, our experience provides valuable clinical insight and lays the groundwork for future multicenter trials. Looking ahead, it will be essential to integrate the bidirectional approach with novel targeted agents (e.g., anti-FGFR2b, Claudin 18.2), immunotherapy, or maintenance strategies to extend the indications to patients without current molecular targets. Additional developments include the use of predictive response systems (PRGS score, cytologic conversion, imaging criteria) and optimization of minimally invasive cytoreductive and HIPEC techniques, which are already under investigation in advanced centers.

## 5. Conclusions

This study highlights the potential benefits of neoadjuvant intraperitoneal treatment in Western patients, supporting its role beyond the Eastern context, where it is already a standard of care. Our findings suggest that NIPS is a safe, well-tolerated, and effective strategy, achieving high rates of conversion surgery comparable to those reported in Eastern countries. Given these promising results, bidirectional chemotherapy should be considered in the preoperative setting for highly selected Western patients with gastric cancer and peritoneal metastases. Future studies should focus on refining multimodal approaches, optimizing patient selection, and integrating novel therapies to further improve survival outcomes.

## Figures and Tables

**Table 1:** Demographic, clinical and histological characteristics of the patients included.

	All Patients	CRS + HIPEC
	N° 25	N° 13
**Age: Median (IQR)**	55 (44–63)	60 (55–63)
**Gender (%)**		
Male	13 (52%)	7 (53.8%)
Female	12 (48%)	6 (46.2%)
**PCI pre-NIPS: (IQR)**	12 (7–12)	9 (6–12)
**Histotype (%)**		
Diffuse	22 (88%)	12 (92.3%)
Intestinal	3 (12%)	1 (7.7%)
**ECOG (%)**		
0	16 (64.0)	8 (61.5)
1	9 (36.0)	5 (38.5)
**Cycles of NIPS (%)**		
3	21 (84%)	12 (92.3%)
>3	4 (16%)	1 (7.7%)
**Adverse Event Toxicity Grade < 3 (%)**		
Abdominal pain	2 (8%)	-
Vomiting	2 (8%)	-
Diarrhea	3(12%)	-
Constipation	4 (16%)	-
Fatigue	8 (32%)	-
**PCI post-NIPS (IQR)**	NA	6 (4–10)
**Adjuvant Chemotherapy (%)**	-	13 (100%)

CRS: cytoreductive surgery; HIPEC: hyperthermic intraperitoneal chemotherapy; PCI: Peritoneal Cancer Index; NIPS: normothermic intraperitoneal and systemic chemotherapy; ECOG: Eastern Cooperative Oncology Group Performance Status.

**Table 2:** Results of conversion surgery.

Variables	Number of Patients N° 13
**D2 + Total gastrectomy**	13 (100%)
**pT**	
pT4a	11 (84.6%)
pT4b	2 (15.4%)
**pN**	
pN0	5 (38.4%)
pN1	2 (15.4%)
pN2	3 (23.1%)
pN3	3 (23.1%)
* Peritonectomy procedures	
Anterior parietal	7 (NA)
Pelvic	6 NA)
Omental bursa	2 NA)
Left upper quadrant	1 (NA)
**Visceral resection**	
Small bowel	3 (23.1%)
Splenectomy	3 (23.1%)
Uterus	2 (15.4%)
Large bowel	2 (15.4%)
**Clavien–Dindo Complication**	
II	2 (15.4%)
IIIB	2 (15.4%)
**Completeness of Cytoreduction**	
CC 0	10 (72.9%)
CC1	3 (23.1%)
**PRGS**	-
1	-
2	13 (100%)
3	-
4	-

PRGS: Peritoneal Regression Grading Score; * The total number exceeds 13 because some patients underwent multiple peritonectomy procedures.

## Data Availability

The data presented in this study are available on request from the corresponding author. The data are not publicly available due to privacy issues.
